# Supplementary material for: Detection of acute promyelocytic leukemia in peripheral blood and bone marrow with annotation-free deep learning
Source: Sci Rep. 2023 Feb 13;13:2562. doi: 10.1038/s41598-023-29160-4 (PMC9925435; doi:10.1038/s41598-023-29160-4)
Supplement: Supplementary file 1 — Supplementary Information. [file 41598_2023_29160_MOESM1_ESM.docx]

Supplementary Material:

**Detection of Acute Promyelocytic Leukemia in Peripheral Blood and Bone Marrow with Annotation-Free Deep Learning**

**Authors:**

Petru Manescu**^1*^**, Priya Narayanan^1^, Christopher Bendkowski**^1^**, Muna Elmi**^1^**, Remy Claveau**^1^**, Vijay Pawar**^1^**, Biobele J. Brown**^2^**, Mike Shaw**^1^**, Anupama Rao**^3^**, and Delmiro Fernandez-Reyes**^1,2*^**

**Affiliations:**

1. Department of Computer Science, Faculty of Engineering Sciences, University College London, Gower Street, London, WC1E 6BT, United Kingdom.
2. Department of Paediatrics, College of Medicine University of Ibadan, University College Hospital, Ibadan, Nigeria.
3. Department of Haematology, Great Ormond Street Hospital for Children, London, WC1N 3JH, United Kingdom.

**Supplementary Table 1.** Comparison of singe-cell classification performance between MILLIE and published fully supervised models.

| **Models** | **Datasets** | | |
| --- | --- | --- | --- |
|  | **AUROC PBS ^[1]^** | **AUROC BMA ^[2]^** | |
|  | **Lymphoblasts** | **Myeloblasts** | **Promyelocytes** |
| Fully Supervised | 0.97 **^[3]^** | 0.874 **^[4]^** | 0.919 **^[4]^** |
| MILLIE (Weakly Supervised) | 0.97 | 0.862 | 0.895 |

The datasets used to train MILLIE do not necessarily contain single-cell level annotations, therefore a comparison using the same training datasets is not possible. Nevertheless, we compared MILLIE with fully supervised approaches by adding a comparison of our cell-level classification performance with results reported in the literature obtained using fully supervised models tested on the same cell-level datasets.

PBS = Peripheral Blood Smears; BMA = Bone Marrow Aspirate

References:

1. Ruggero Donida Labati, Vincenzo Piuri FS. ALL-IDB: THE ACUTE LYMPHOBLASTIC LEUKEMIA IMAGE DATABASE FOR IMAGE PROCESSING. Ieee Int. Conf. Image Process. 2011;2089–2092.
2. Eckardt JN, Middeke JM, Riechert S, et al. Deep learning detects acute myeloid leukemia and predicts NPM1 mutation status from bone marrow smears. Leukemia. 2021;(August)
3. Schouten *et al.* Tens of images can suffice to train neural networks for malignant leukocyte detection, Scientific Reports, 2021. <https://www.nature.com/articles/s41598-021-86995-5>
4. Eckardt JN, Schmittmann T, Riechert S, et al. Deep learning identifies Acute Promyelocytic Leukemia in bone marrow smears. BMC Cancer. 2022;22(1):1–11.

**Supplementary Figure 1.** Feature fusion strategy comparison of sample level classification performance. **a.** Comparison between Max Pooling and Attention Pooling on the PBS dataset (one run) (Fig. 3). **b.** Comparison between Max Pooling and Attention Pooling on the BMA dataset (one run) (Fig. 5).
